# PPARγ alleviates preeclampsia development by regulating lipid metabolism and ferroptosis

**DOI:** 10.1038/s42003-024-06063-2

**Published:** 2024-04-09

**Authors:** Weisi Lai, Ling Yu, Yali Deng

**Affiliations:** Department of Obstetrics and Gynecology, Second XiangYa Hospital of Central South University, Changsha, China

**Keywords:** Mechanisms of disease, Reproductive signs and symptoms

## Abstract

The study aims to explore the effect of PPARγ signaling on ferroptosis and preeclampsia (PE) development. Serum and placental tissue are collected from healthy subjects and PE patients. The PPARγ and Nrf2 decreases in the PE. Rosiglitazone intervention reverses hypoxia-induced trophoblast ferroptosis and decreases lipid synthesis by regulating Nfr2 and SREBP1. Compared to the Hypoxia group, the migratory and invasive abilities enhance after rosiglitazone and ferr1 treatment. Rosiglitazone reduces the effect of hypoxia and erastin. The si-Nrf2 treatment attenuats the effects of rosiglitazone on proliferation, migration, and invasion. The si-Nrf2 does not affect SREBP1 expression. PPARγ agonists alleviates ferroptosis in the placenta of the PE rats. The study confirms that PPARγ signaling and ferroptosis-related indicators were dysregulated in PE. PPARγ/Nrf2 signaling affects ferroptosis by regulating lipid oxidation rather than SREBP1-mediated lipid synthesis. In conclusion, our study find that PPARγ can alleviate PE development by regulating lipid oxidation and ferroptosis.

## Abstract (web summary)

Hypertension, and organ system dysfunctions associated with preeclampsia (PE) result in poor pregnancy outcomes. In the current study, the authors examined molecular pathogenesis of PE with a focus Peroxisome proliferator-activated receptor gamma (PPARγ), lipid metabolism, and ferroptosis in the placenta of PE patients.

## Introduction

Preeclampsia (PE), a pregnancy-specific disorder, is the leading cause of maternal and perinatal morbidity and mortality worldwide, accounting for approximately 2–5% of all^1^. The occurrence of PE is often accompanied by hypertension and proteinuria. In severe cases, multiple organ system damage or failure and even death may occur^2,3^. Most current theories support that the onset of PE is a two-stage process^1,4,5^. Stage 1 represents placental dysfunction due to insufficient remodeling of the spiral arteries due to superficial trophoblast invasion. This leads to impaired blood exchange and inadequate oxygen supply, which promotes oxidative stress. In the second stage, inflammatory factors produced by syncytiotrophoblast stress lead to systemic vascular inflammation and endothelial dysfunction, resulting in maternal clinical symptoms of hypertension and organ failure. Currently, the clinical treatment of PE is limited, focusing on controlling acute hypertension, preventing eclampsia, and timely delivery^6,7^. Therefore, exploring the pathogenesis of PE can guide the prevention and treatment of PE in clinical practice, thereby improving maternal pregnancy outcomes.

Lipids, as structural components of cell membranes, signaling mediators, and energy reservoirs, can reflect the physiological or pathological states of metabolic diseases such as PE^8^. The lipid metabolism of the placenta and serum of women with PE was altered, and the composition of lipid metabolism might indicate the pathogenesis of PE^8–10^. Lipid profiles, including total cholesterol (T-CHO) and triglyceride (TG), can be screening markers for dyslipidemia. As pregnancy progresses, properly elevated blood lipids can provide proper nutrition for the fetus^11^. Prenatal serum TG and free fatty acid concentrations in PE were approximately twofold higher than those in uncomplicated women, well above the normal range^12^. Furthermore, changes in lipid profile were also significantly correlated with blood flow in the PE uterus^13^. Ferroptosis is an iron-dependent oxidative, non-apoptotic death pathway discovered in recent years and related to the imbalance of cellular lipid peroxidation and redox capacity^14^. Studies have found that ferroptosis is related to neurological diseases, cardiovascular and cerebrovascular diseases, and cancer^15–17^. Excess iron intake or high iron status during pregnancy can affect placental blood flow, maternal inflammation, and infection, potentially leading to adverse delivery outcomes^18,19^. However, the intrinsic mechanism of lipid metabolism and ferroptosis involved in developing PE is still unclear and needs further exploration.

Peroxisome proliferator-activated receptor gamma (PPARγ) is a member of the nuclear receptor family of ligand-activated transcription factors and can heterodimerize with retinoic acid X receptor (RXR) to regulate gene expression^20^. PPARγ regulates the body’s glucose and lipid metabolism, inflammation, oxidative stress, and insulin resistance^21^. Studies have shown that PPARγ could play an important role in trophoblast differentiation and placental development, and the expression level of PPARγ was closely related to the pathogenesis of PE^22,23^. Nonetheless, the role of PPARγ in PE trophoblasts has not been fully elucidated.

In this study, we found that PPARγ was associated with lipid metabolism and ferroptosis in the placenta of PE patients. Regulation of PPARγ could affect hypoxia-induced ferroptosis, possibly due to PPARγ targeting Nrf2 signaling to regulate lipid peroxidation. The mechanistic diagram is shown in Fig. S1.

## Methods

### Participant information

The serum and placental tissue samples from healthy pregnant women (Normal) and patients with PE were obtained from the Second Xiangya Hospital of Central South University, 20 in each group. The study was approved by Second Xiangya Hospital of Central South University Ethics Committee (2020-584). All subjects were given informed consent.

PE was diagnosed and classified according to the criteria provided by the International Society for the Study of Hypertension in Pregnancy (ISSHP)^2^. As described in a previous study^24^, the inclusion criteria for the Normal were normotensive during pregnancy, term pregnancy, no history of chronic metabolic disease or any pathology that may involve disturbances in lipid or carbohydrate metabolism, and no complications during pregnancy. Exclusion criteria for PE were patients with cardiovascular disease, diabetes, metabolic syndrome, infection, congenital malformations, and chromosomal abnormalities (number or structure). The subjects’ clinical baseline information is shown in Table 1.

**Table 1:** Subject baseline information statistics table

	Normal	PE	*p* value
	20	20	
Ethnic Group	Han	Han	
Age (years)	32.3 ± 5.26	32.55 ± 5.37	0.8825
Pregravid body mass index (kg/m^2^)	21.85 ± 3.23	23.98 ± 6.10	0.1741
Blood pressure (mmHg)			
SBP	126.25 ± 6.93	172.25 ± 21.24	<0.0001
DBP	78.8 ± 6.49	106.65 ± 11.94	<0.0001
Gestational age (weeks)	38.54 ± 1.79	34.38 ± 2.8	<0.0001
Parity			>0.9999
Primigravid	11	10	
Multiparous	9	10	
Neonatal sex			>0.9999
Male	8	9	
Female	12	11	
Neonatal birth weight (g)	3144.5 ± 573.82	2087.3 ± 736.08	<0.0001

Data statistics were performed using unpaired *t*-test and Chi-square.

*SBP* systolic blood pressure, *DBP* diastolic blood pressure.

### Cell culture and treatment

HTR-8/SVneo cells were obtained from Shanghai Zhong Qiao Xin Zhou Biotechnology Co., Ltd. Cells were cultured in RPMI-1640 medium containing 10% fetal bovine serum (FBS) and 1% penicillin/streptomycin. Cells were grown at 37 °C, 5% CO_2_.

The si-PPARγ, si-RXRα, si-Nrf2, oe-GPX4, and negative controls (si-NC and oe-NC) were purchased from HonorGene (Changsha, China). The si-PPARγ, si-RXRα, si-Nrf2, oe-GPX4, si-SREBP1, and negative controls were transfected into cells by Lipofectamine 2000. Rosiglitazone (122320-73-4, MedChemExpress, USA) is an agonist of PPARγ. Erastin (HY-15763, MedChemExpress, USA) is an agonist of ferroptosis and ferrostatin-1 (ferr1, HY-100579, MedChemExpress, USA) is an inhibitor. After treatment with 1 μM rosiglitazone, 1 μM ferr1, and 1 μmol erastin for 24 h, cells were subjected to hypoxia for 24 h. As described earlier^25^, the cells were placed under 2% O_2_ for 24 h to establish a hypoxic cell model. Cells in the NC group were given normoxic conditions with 20% O_2_. Cells were observed by a transmission electron microscope (jem-1400).

### Determination of TG, T-CHO, low-density lipoprotein cholesterol (LDL), high-density lipoprotein cholesterol (HDL), malondialdehyde (MDA), glutathione (GSH), Fe(II), and urine protein levels

Serum, placental tissue, and cell samples were collected, and total protein was extracted. Bicinchoninic Acid Assay (BCA, HG-WDP0003a, HonorGene, China) was performed to detect total protein content. Concentrations of TG, T-CHO, LDL, HDL, MDA, GSH, Fe(II), and urine protein were measured by commercial kits, following the manufacturer’s instructions. Detection kits for TG (A110-1-1), T-CHO (A111-2-1), LDL (A113-1-1), HDL (A112-1-1), MDA (A003-1), GSH (A006-1-1), and urine protein (C035-2-1) were purchased from Nanjing Jiancheng Bioengineering Institute. Fe(II) was measured by an iron detection kit (TC1015, Beijing Leagene Biotechnology). A microplate reader was operated to measure optical density (OD) values.

### Western blot

Total protein from placental tissue and cells was extracted by radioimmunoprecipitation (RIPA) butter. Nuclear and cytoplasmic proteins were extracted using the Nucl-Cyto-Mem Preparation Kit (P1201-50, APPLYGEN, China). The BCA method was operated to detect protein concentration. 10% and 12% gels were used for SDS-polyacrylamide gel electrophoresis. Proteins were transferred from the gel to nitrocellulose membranes and blocked with 5% nonfat milk overnight at 4 °C. The primary and secondary antibodies were incubated for 90 min each. After adding SuperECL Plus supersensitive luminescent solution (AWB0005, abiowell, China), a chemiluminescence imaging system (ChemiScope 6100) was operated for visualization. The anti-PPARγ (16643-1-AP, 1:6000), anti-Nrf2 (16396-1-AP, 1:5000), anti-SREBP1 (14088-1-AP, 1:2000), anti-FASN (10624-2-AP, 1:2000), anti-ACC1 (21923-1-AP, 1:4000), anti-GPx4 (67763-1-Ig, 1:5000), anti-SLC7A11 (26864-1-AP, 1:1000), anti-FPN1 (26601-1-AP, 1:300), anti-FTH1 (10727-1-AP, 1:5000), anti-TFR1 (66180-1-Ig, 1:20000), anti-CyclinD1 (60186-1-Ig, 1:2000), anti-BCL2 (12789-1-AP, 1:2000), anti-C-Myc (10828-1-AP, 1:6000), anti-pHH3 (ab5176, 1:5000), anti-MMP2 (10373-2-AP, 1:800), anti-MMP9 (10375-2-AP, 1:1000), anti-TIMP-1 (16644-1-AP, 1:3000), anti-TIMP-2 (17353-1-AP, 1:2000), anti-β-actin (66009-1-Ig, 1:5000), anti-PCNA (10205-2-AP, 1:5000), anti-mouse IgG (SA00001-1, 1:5000), anti-rabbit IgG (, 1:6000) were purchased from proteintech (USA). The anti-SCD1 (ab19862, 1:1000) and anti-TFR2 (ab80194, 1 µg/ml) were purchased from abcam (UK). The anti-ACSL4 (AWA41262, 1:1000) was purchased from Abiowell (China). β-actin and PCNA were used as a reference standard for protein content. Uncropped blots are shown in Fig. S2.

### Immunohistochemistry (IHC)

Placental tissue was collected and fixed. Samples were cut into 5 μm paraffin sections. Sections were immersed in 0.01 M citrate buffer (pH 6.0) and heated continuously for 20 min. 1% periodic acid was used to inactivate endogenous enzymes. Sections were dripped with primary antibody and left overnight at 4 °C. A dilution ratio of 1:200 was used for PPARγ and Nrf2. After that, the sections were incubated with 60 μl of secondary antibody and incubated at 37 °C for 30 min. 50 μl of diaminobenzidine (DAB) working solution was added, and the reaction was carried out for 3 min. After hematoxylin and PBS treatment, various alcohols (60–100%) were used for dehydration. After treatment with dimethylbenzene and neutral resin, the samples were observed and photographed under a microscope.

### Detection of 8-hydroxy-2-deoxyguanosine (8-OHdG) levels

Placental tissue, cell, and serum samples were collected. Total protein was extracted, and its content was determined using the BCA kit. The human 8-OHdG ELISA kit was obtained from CUSABIO (China). Levels of 8-OHdG were determined according to the manufacturer’s instructions. A microplate reader was operated to determine the OD value.

### ROS levels

After fresh tissue was washed 3 times with 5 ml PBS, the tissue was minced to a paste with ophthalmic scissors. 5 ml of collagenase Type I was added to digest for 40 min at 37 °C on a shaker. 5 ml complete Dulbecco’s modified eagle medium (DMEM) was added to stop digestion. The cell suspension was collected and centrifuged at 1500 rpm for 5 min to obtain cell pellets. After the cells were resuspended in 5 ml of erythrocyte lysate, the samples were allowed to stand at room temperature for 5 min, and then centrifuged at 1500 rpm for 5 min to obtain cell pellets. After resuspending and washing with 5 ml PBS, 2 ml of DMEM medium was added. The BODIPY™ 581/591 C11 (D3861, Thermofisher, Italy) was used to analyze lipid ROS levels, following the manufacturer’s instructions. Flow cytometry was performed to detect fluorescence.

Levels of lipid ROS in the cells were analyzed by C11-BODIPY staining. The BODIPY™ 581/591 C11 working solution was added to the cells and incubated for 30 min at room temperature. After washing, the cells were subjected to DAPI staining. After incubation for 20 min, images were taken and observed.

### Prediction of PPARγ binding to Nrf2

The online software STRING was used to predict the binding of PPARγ to SREBP1 and Nrf2.

Molecular docking was performed to analyze the potential binding sites between PPARγ and Nrf2. The structures of PPARγ and Nrf2 were obtained from the PDB database (https://www.rcsb.org/). These protein were prepared by adding hydrogen atoms and manizing energy. ZDOCK3.0 algorithm was used to predict protein-protein complex structures. ZDOCK separates the full six-dimensional rigid-body space into a three-dimensional translational space and a three-dimensional rotational space. The best conformation were obtained and visualized using PyMOL (https://pymol.org/2/).

### Co-Immunoprecipitation (Co-IP)

IP cell lysis buffer (AWB0144, abiowell, China) was used to extract total cell proteins. The samples were divided into Input, IgG, and PPARγ-IP. Rabbit IgG (B900610, proteintech, USA) and anti-PPARγ were added to the IgG and the PPARγ-IP groups, respectively. After overnight incubation at 4 °C with rotating mixing, pretreated agarose beads were added for immunoprecipitation. The IP cell lysate was used to wash excess antibodies and repeated 4 times. The coupled proteins on the agarose gels were separated in a boiling water bath. Proteins in the Input group were not processed. The expressions of PPARγ, Nrf2 and SREBP1 were detected by western blot. Uncropped blots are shown in Figure S2.

### Real-time quantitative PCR (qRT-PCR)

Total RNA from tissues and cells was extracted by the Trizol method. The total mRNA was used as the template, and cDNA was reverse transcribed using a HiFiScript cDNA Synthesis Kit (CW2569, CWBIO, China). UltraSYBR Mixture (CW2601, CWBIO, China) was used for PCR amplification. Primers of the target gene were designed using primer5 software and purchased from Tsingke Biotechnology Co., Ltd. 2^−ΔΔCt^ was used to calculate the relative expression of genes. β-actin was used as control data. The primer sequences are shown in Table 2.

**Table 2:** The primer sequences for PCR

Gene name	Primer sequences	Primer length
β-actin	Forward: ACCCTGAAGTACCCCATCGAG	224 bp
	Reverse: AGCACAGCCTGGATAGCAAC	
GPX4	Forward: CGCCTTTGCCGCCTACTGAAGC	150 bp
	Reverse: AACCATGTGCCCGTCGATGTCC	

### Cell counting kit 8 (CCK8) assay

Cell proliferation was evaluated using the CCK-8 (Nu679, Dojindo, Japan) according to the manufacturer’s instructions. Cells were plated into 96-well plates with 5 × 10^3^ cells per well and cultured for 24 h. 100 μl of CCK-8 working solution was added for 4 h. The OD value at 450 nm was measured by a microplate reader.

### Wound healing

Cells were added to 6-well plates at 5 × 10^5^ cells/well. A 10 µl pipette tip was used to scrape the cells in the plate. Images were acquired at different times (0 h and 48 h) after wounding.

### Transwell assay

The transwell inserts were coated with 100 μl of 2 mg/ml Matrigel matrix at 37 °C for 30 min. Cells resuspended in serum-free medium at 2 × 10^4^ cells/well were added to the upper chamber. 500 μl medium containing 10% FBS was added to the lower chamber. After incubation for 48 h at 37 °C, excess cells in the upper chamber were removed with a cotton swab, and cells were fixed with 4% paraformaldehyde for 20 min. 0.1% crystal violet solution was used for staining for 5 min. Cells were observed and counted under a microscope.

### Animals experiences

All animal experiments were approved by the Animal Ethical and Welfare Committee, the Second Xiangya Hospital, Central South University, China (2021806). SD rats (250 ± 10 g) were purchased from Hunan sta Laboratory Animal Co., Ltd. All animals were adaptively fed for one week with a 12 h light/dark cycle and free access to food and water.

Healthy adult rats were selected, and male and female rats were caged at a ratio of 2:1 at night. Rat vaginal secretion smears were taken every morning, and the day when sperm was detected under a light microscope was defined as the 0th day of pregnancy. Pregnant rats were randomly divided into 3 groups (5 rats in each group) on the 14th day of gestation: Control group, PE group, and PE + rosiglitazone group. On day 14, PE animal models were established by reducing uterine perfusion pressure (RUPP) in the PE and PE + rosiglitazone group, as described in our previous study^26^. Pregnant rats in the control group were subjected to sham surgery. After the operation, the pregnant rats were supplemented with an appropriate amount of penicillin to fight infection, kept warm, and routinely fed. Pregnant rats in the PE + rosiglitazone group were given 5 mg/kg rosiglitazone by gavage on the second day after modeling for 5 days^27^. On the 14th, 16th, and 19th day of gestation, the systolic blood pressure (SBP) and diastolic blood pressure (DBP) levels were detected. Serum, urine and placenta were collected after the blood pressure test on day 19 of pregnancy. Hematoxylin and eosin (H&E) staining was used to observe placental changes.

### Statistics and reproducibility

All statistical analyses were conducted using GraphPad Prism 8.0.1. Data are presented as mean ± SD. Data comparisons between the two groups were performed with an unpaired *t*-test. One-way ANOVA and two-way ANOVA analyze data between multiple groups. Statistical analysis of categorical variables was performed by chi-square test. Pearson correlation was performed, and *P* value < 0.05 was considered statistically significant. At least three biological replicates were performed for each experiment.

### Reporting summary

Further information on research design is available in the Nature Portfolio Reporting Summary linked to this article.

## Results

### PPARγ and Nrf2 might be linked to the ferroptosis pathway in PE

**Fig. 1:**
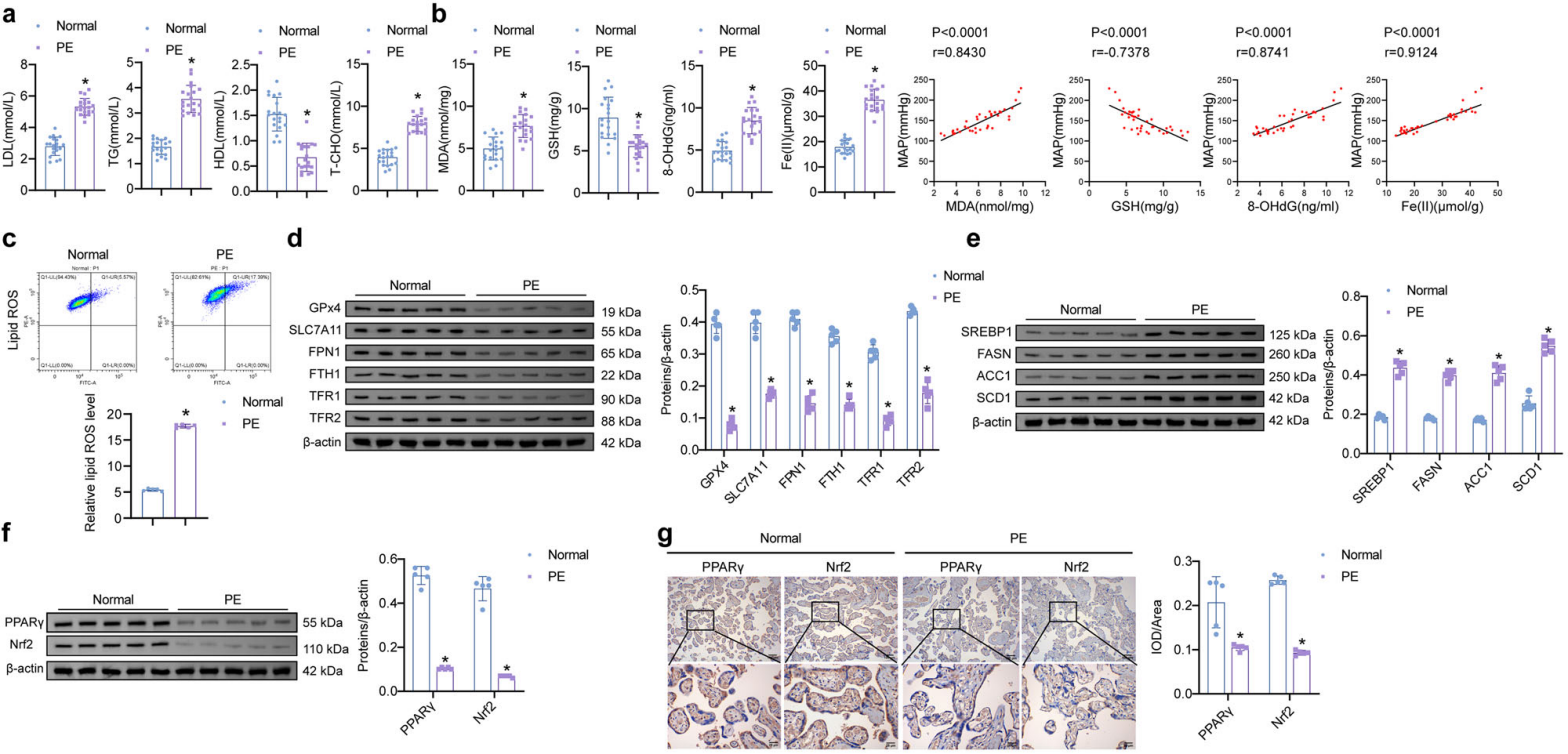
The expression of ferroptosis-related indicators, PPAPγ, and Nrf2 is changed in PE. **a** Levels of lipid metabolites (LDL, TG, HDL, and T-CHO) in serum. **b** Contents of MDA, GSH, 8-OHdG, and Fe(II) in serum and the correlation with MAP. (*n* = 20). **c** Lipid ROS levels in placental tissue. **d** Protein expression of ferroptosis pathway-related indicators (GPx4, SLC7A11, FPN1, FTH1, TFR1, and TFR2). **e** Western blot detection of lipid synthesis SREBP1 pathway-related indicators (SREBP1, FASN, ACC1, and SCD1). **f** Protein expression of PPAPγ and Nrf2 in placental tissue. (*n* = 5). **g** Levels of PPAPγ and Nrf2 were detected by IHC in placental tissue. Brown represents the positive expression of PPARγ or Nrf2. (100× and 400×, *n* = 5). Data represent the mean ± SEM. *P < 0.05 vs the Normal group, unpaired *t*-test.

Serums of normal and PE patients were collected. The contents of lipid metabolites (LDL, TG, HDL, and T-CHO) were altered. LDL, TG, and T-CHO levels increased in PE patients compared with the Normal, while HDL decreased (Fig. 1a). Meanwhile, MDA, 8-OHdG, and Fe(II) were higher in the PE than in the normal groups (Fig. 1b). GSH levels were the opposite. Pearson correlation analysis showed that MDA, 8-OHdG, and Fe(II) levels were positively correlated with mean artery pressure (MAP) (*P* < 0.0001). There was a negative correlation between GSH and MAP (*P* < 0.0001) (Fig. 1b). Lipid ROS levels were significantly higher in the PE than in the normal groups (Fig. 1c). It was speculated that the development of eclampsia may be related to ferroptosis.

Ferroptosis pathway-related indicators (GPx4, SLC7A11, FPN1, FTH1, TFR1, and TFR2) was further detected. The expression of GPx4, SLC7A11, FPN1, FTH1, TFR1, and TFR2 decreased in PE (Fig. 1d). The protein levels of lipid synthesis SREBP1 pathway-related indicators (SREBP1, FASN, ACC1, and SCD1) were determined. Compared with the Normal group, SREBP1, FASN, ACC1, and SCD1 in placental tissue increased in the PE group (Fig. 1e). The expression of Nrf2 is related to PPARγ regulation^28^. The protein levels of PPAPγ and Nrf2 in placental tissue were lower in PE than in the healthy (Fig. 1f, g). Results of correlation analysis (Fig. S3) showed that levels of PPARγ might be linked to Nrf2 and ferroptosis-related indicators in PE.

### The PPARγ agonist protects against hypoxia-induced trophoblast ferroptosis and decreased lipid synthesis

**Fig. 2:**
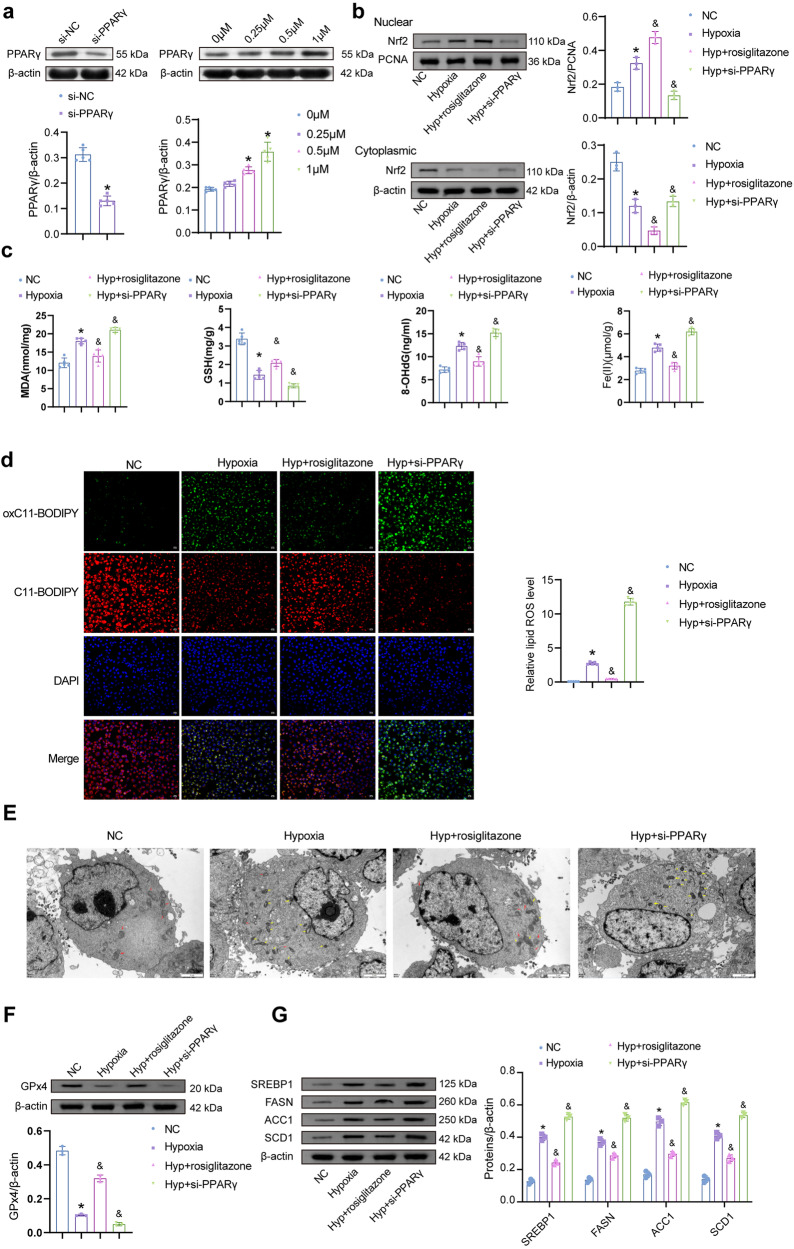
PPARγ promotes the expression of Nrf2 and affects ferroptosis and the process of lipid synthesis. **a** PPARγ protein expression was identified after PPARγ siRNA intervened. (*n* = 5). Data represent the mean ± SEM. *P < 0.05 vs the si-NC group, unpaired *t*-test. PPARγ protein expression was identified after rosiglitazone intervened. (*n* = 3) *P < 0.05 vs the 0 μM group, one-way ANOVA. **b** The protein levels of Nrf2 (nuclear and cytoplasmic). (*n* = 3). **c** The concentrations of MDA, GSH, 8-OHdG, and Fe(II) were identified. **d** Levels of lipid ROS. (200×, *n* = 5). **E** To determine whether the cells underwent ferroptosis, the transmission electron microscope was performed. Yellow arrows indicate that mitochondria are characterized by ferroptosis, as shown by thickening of mitochondrial crista structure, thickening of mitochondrial membrane, and mitochondrial shrinkage. The red arrows indicate normal mitochondrial characteristics, and the cristae structure is neatly arranged and relatively intact. (8000×). **F** Protein levels of GPx4. **G** The expression of lipid synthesis-related markers SREBP1, FASN, ACC1, and SCD1 was determined. (*n* = 3). Data represent the mean ± SEM. *P < 0.05 vs the NC group, ^&^*P* < 0.05 vs the Hypoxia group, one-way ANOVA.

HTR-8/Svneo cells were intervened using PPARγ siRNA or different concentrations of the PPARγ agonist rosiglitazone (0, 0.25, 0.5, and 1 μM). The protein expression of PPARγ was lower in the si-PPARγ than in the si-NC groups. This indicated that PPARγ siRNA was successfully transfected. Levels of PPARγ increased with increasing concentrations of rosiglitazone (Fig. 2a). Thus, 1 μM rosiglitazone was employed in subsequent experiments. The rosiglitazone is also an inhibitor of ACSL4^29^. However, levels of ACSL4 were not significantly different in the rosiglitazone concentration range of 0–1 μM (Fig. S4a). These results indicated that rosiglitazone (0–1 μM) mainly activated PPARγ but had no effect on ACSL4 in HTR-8/Svneo cells.

HTR-8/Svneo cells were cultured, and an in vitro model of PE was constructed under hypoxic conditions. PPARγ siRNA and rosiglitazone were used to intervene in the cells. Nrf2 (nuclear) levels increased in the Hypoxia compared to the NC groups. After rosiglitazone intervention, the protein levels of Nrf2 (nuclear) increased compared with the Hypoxia group. Meanwhile, Nrf2 (nuclear) levels were lower in the hypoxia (Hyp) + si-PPARγ group than in the Hypoxia (Fig. 2b). It indicated that PPARγ could affect the nuclear translocation level of Nrf2. The contents of MDA, GSH, 8-OHdG, and Fe(II) were further detected. MDA, 8-OHdG, and Fe(II) levels in the Hypoxia group were higher than in the NC group and decreased after rosiglitazone intervention (Fig. 2c). The expression of GSH was reversed. The lipid ROS in the Hyp + rosiglitazone group was lower than that in the Hypoxia (Fig. 2d). After silencing PPARγ, its content increased. Mitochondrial membrane thickening and mitochondrial shrinkage were observed in cells treated with hypoxia and PPARγ silencing (Fig. 2E). The relative number of mitochondria characteristic of ferroptosis decreased in rosiglitazone treated cells. The protein level of GPx4 was elevated in the Hyp + rosiglitazone group compared to the Hypoxia (Fig. 2F). These suggested that PPARγ could suppress hypoxia-induced ferroptosis. Levels of lipid synthesis-related indicators SREBP1, FASN, ACC1, and SCD1 were higher in the Hypoxia group than in the NC group. Moreover, they decreased after rosiglitazone intervention. Silencing PPARγ increased SREBP1, FASN, ACC1, and SCD1 (Fig. 2G). These suggested that PPARγ could regulate the expression of SREBP1, FASN, ACC1, and SCD1, thereby affecting lipid synthesis.

### The effect of PPARγ agonist on proliferation and migration in reversal of hypoxia may be related to the inhibition of cell ferroptosis

**Fig. 3:**
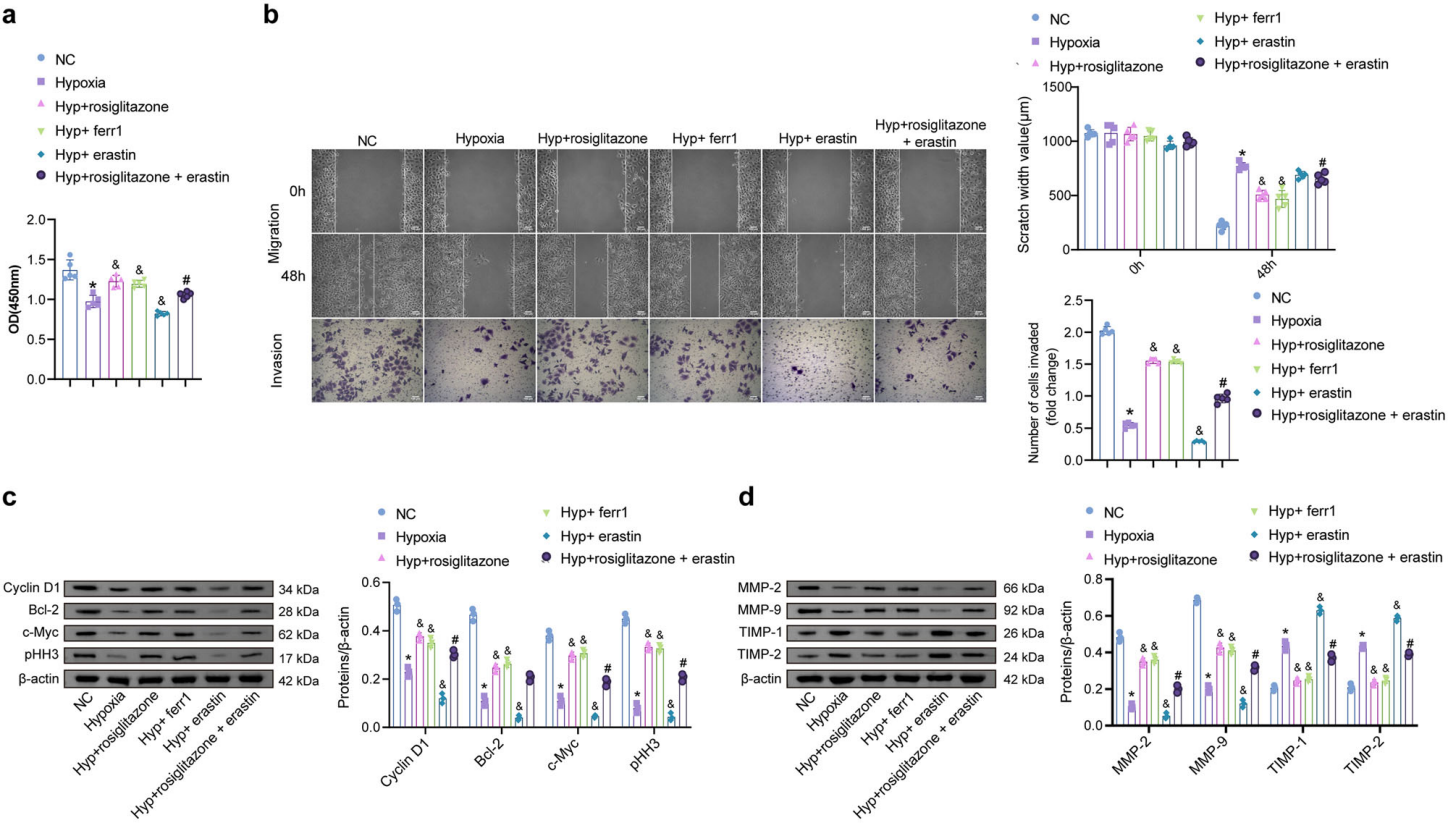
PPARγ may affect the proliferation and migration activities of HTR-8/Svneo cells via ferroptosis. **a** The cell proliferation ability. (*n* = 5). **b** The migration and invasion abilities were analyzed by wound healing and Transwell assay, respectively. In the wound healing experiment, lines were used to standardize the width of the scratch. In the Transwell assay, the invasive cells were stained purple with crystal violet. (100×, *n* = 5). **c** Expression of proliferation-related markers (Cyclin D1, Bcl-2, c-Myc, and pHH3). **d** Protein levels of migration and invasion-related markers (MMP-2, MMP-9, TIMP-1, and TIMP-2). (*n* = 3). Data represent the mean ± SEM. *P < 0.05 vs the NC group, ^&^*P* < 0.05 vs the Hypoxia group, ^#^*P* < 0.05 vs the Hyp + rosiglitazone + erastin group, one-way ANOVA and two-way ANOVA.

Proliferative and migratory activities of HTR-8/Svneo cells were assessed after hypoxia, rosiglitazone, ferr1, and erastin intervention. Hypoxia treatment reduced the proliferative activity of HTR-8/Svneo cells. Compared to the Hypoxia group, cell proliferation was elevated in the Hyp + rosiglitazone and Hyp + ferr1 groups, while lower in the Hyp + erastin group (Fig. 3a). Activation of PPARγ could reverse the hypoxia-induced decrease in proliferative activity. Wound healing and Transwell assays were performed to characterize the migratory and invasive abilities (Fig. 3b). The migratory capacity increased after rosiglitazone and ferr1 intervention, alleviating the hypoxia-induced decrease. Rosiglitazone reversed the reduced migration and invasion ability caused by hypoxia and erastin interventions. The expression of proliferation, migration, and invasion-related markers were detected by Western blot (Fig. 3c, d). Rosiglitazone and ferr1 intervention increased Cyclin D1, Bcl-2, c-Myc, and pHH3 protein levels, and erastin decreased. MMP-2 and MMP-9 were elevated in the Hyp + rosiglitazone and Hyp + ferr1 groups compared to the Hypoxia group. Meanwhile, rosiglitazone could reduce the intervention effect of erastin. The expression of TIMP-1 and TIMP-2 was opposite to that of MMP-2 and MMP-9.

Combined with the above analysis, intervention of ferroptosis could affect the proliferation and migration activity of HTR-8/Svneo cells, and PPARγ may regulate the proliferation and migration of HTR-8/Svneo cells by affecting ferroptosis.

### PPARγ regulates SREBP1 and Nfr2 signaling pathways

**Fig. 4:**
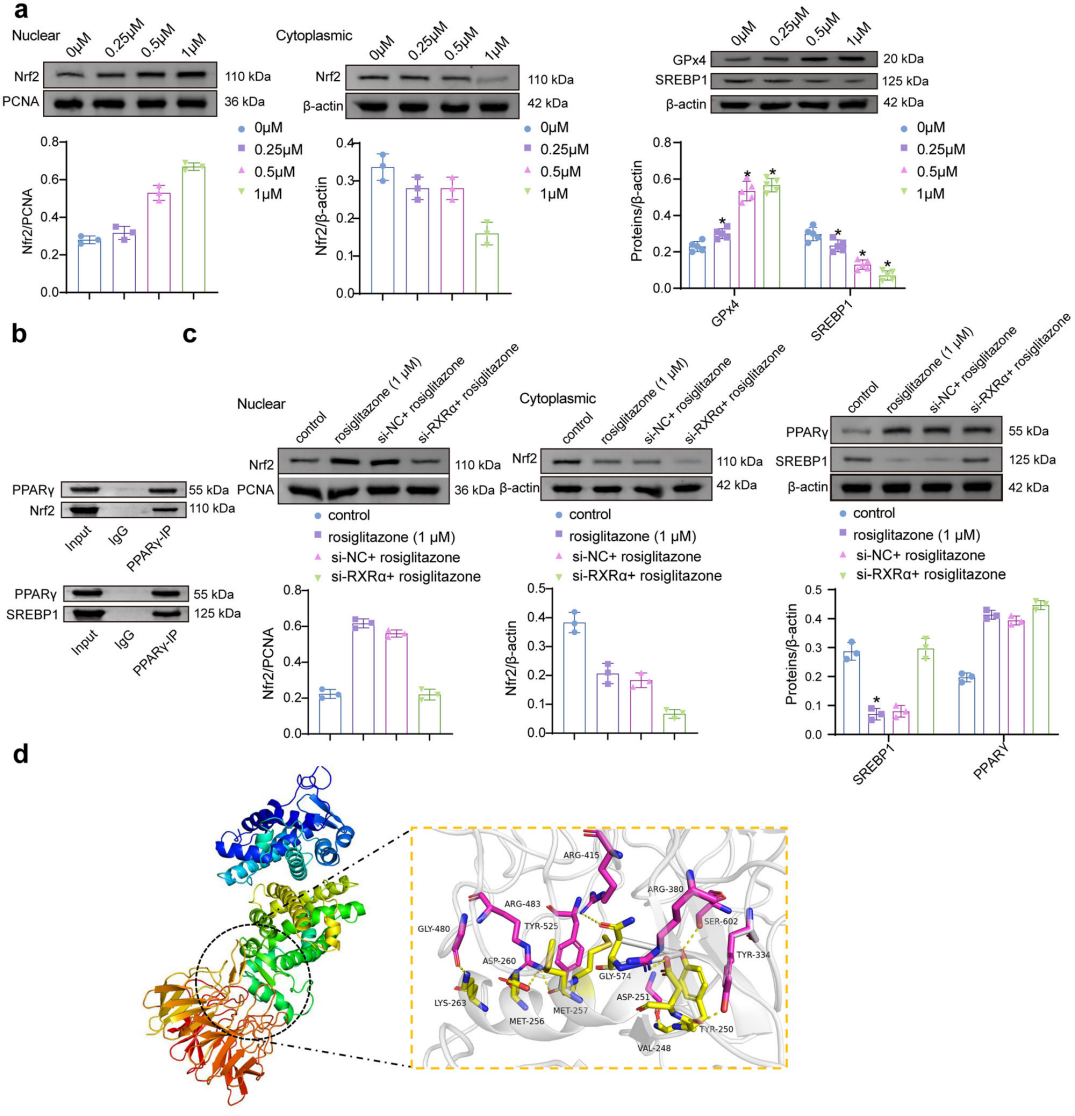
PPARγ directly acts on Nfr2 and SREBP1. **a** Levels of Nrf2 (nuclear and cytoplasmic), GPx4, and SREBP1 were detected by Western blot after rosiglitazone (0, 0.25, 0.5, and 1 μM) intervened in HTR-8/Svneo cells. (*n* = 3). Data represent the mean ± SEM. *P < 0.05 vs the 0 μM group, one-way ANOVA. **b** The interaction was identified between PPARγ and Nrf2 and between PPARγ and SREBP1. **c** The expression of PPARγ, Nrf2 (nuclear and cytoplasmic), and SREBP1. (*n* = 3). Data represent the mean ± SEM. *P < 0.05 vs the si-NC + rosiglitazone group, unpaired *t*-test. **d** The potential binding sites of PPARγ and Nrf2 proteins were predicted using molecular docking.

PPARγ binding to SREBP1 and Nrf2 was predicted using the online software STRING. There may be pairwise interactions among PPARγ, SREBP1, and Nrf2 (Fig. S4b). HTR-8/Svneo cells were intervened with different concentrations of PPARγ agonist rosiglitazone (0, 0.25, 0.5, and 1 μM). The expression of Nrf2, GPx4, and SREBP1 were detected by Western blot. The expression of Nrf2 (nuclear) and GPx4 increased dose-dependent with rosiglitazone, while SREBP1 decreased (Fig. 4a). Co-IP experiments showed that PPARγ/Nrf2 and PPARγ/SREBP1 directly interacted (Fig. 4b). The study has shown that the regulation of PPARγ on downstream target genes is inseparable from the effect of PPARγ/RXRα heterodimer^20^. We performed si-RXRα treatment concurrently with rosiglitazone intervention. Levels of PPARγ, Nrf2, and SREBP1 were detected by Western blot. Nrf2 (nuclear) decreased in the si-RXRα + rosiglitazone group compared to the si-NC + rosiglitazone, while SREBP1 increased (Fig. 4c). We further predicted the potential binding sites of PPARγ and Nrf2 proteins using molecular docking (Fig. 4d). As shown in the docking results, LYS 263, MET 256, MET 257, ASP 251, ASN 253, VAL 248, and TYR 250 of PPARγ protein may form hydrogen bonds with GLY 480, ASP 260, ARG 415, GLY 574, ARG 380, SER 602, and TYR 334 of Nrf2 protein.

### Silencing Nrf2 reverses the protective effect of PPARγ agonists on hypoxia-induced ferroptosis

**Fig. 5:**
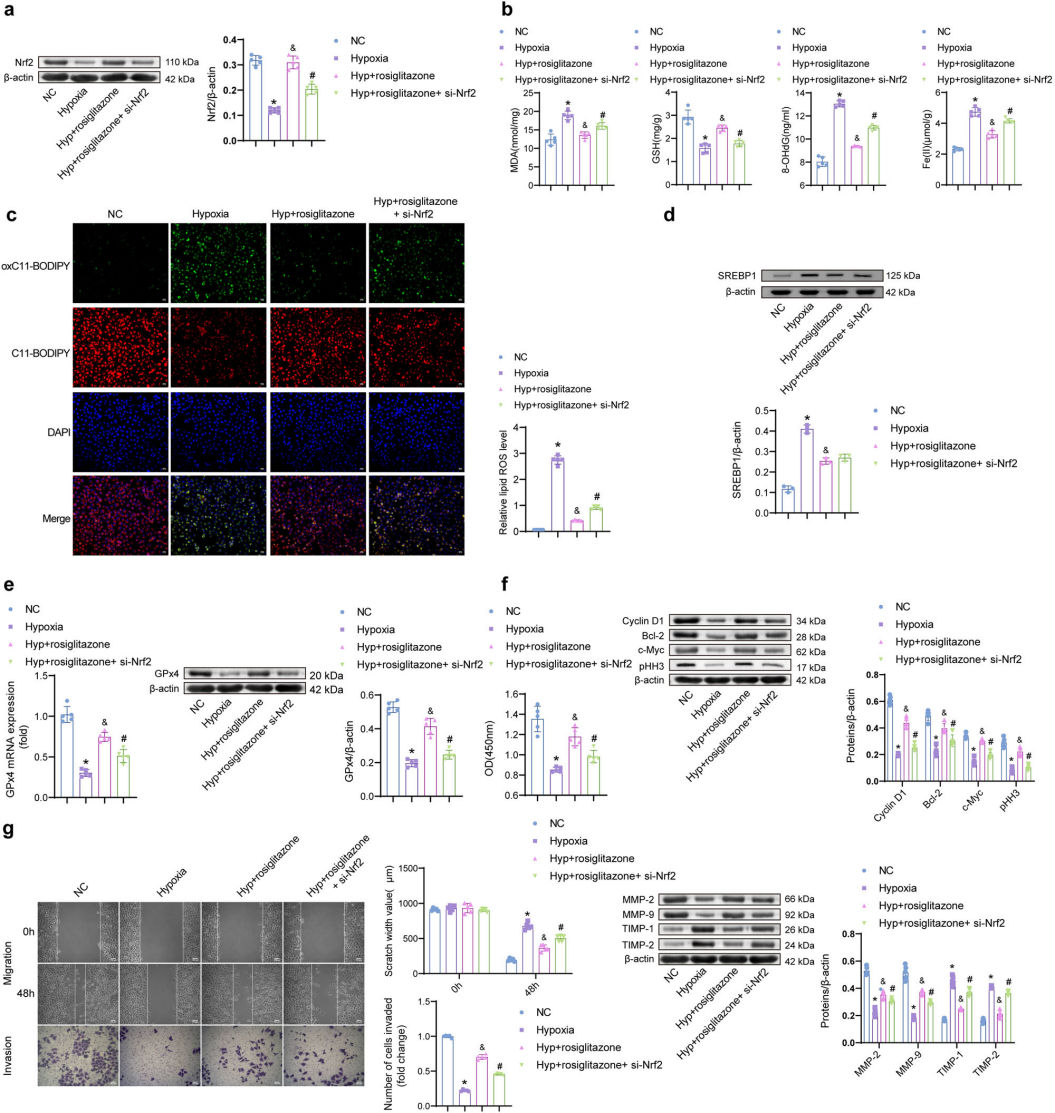
PPARγ regulates hypoxia-induced cellular ferroptosis via Nrf2. **a** Protein expression of Nrf2. **b** Levels of MDA, GSH, 8-OHdG, and Fe(II). (*n* = 5). **c** The lipid ROS levels. (200×, *n* = 5). **d**, **e** The expression of SREBP1 and GPx4. (*n* = 3). **f** CCK-8 detected the cell proliferation activity, and the protein levels of Cyclin D1, Bcl-2, c-Myc, and pHH3 were detected by Western blot. **g** The wound healing and Transwell assay for cell migration and invasion activity (100×, *n* = 5) and Western blot (*n* = 5) for MMP-2, MMP-9, TIMP-1, and TIMP-2 protein levels. Data represent the mean ± SEM. *P < 0.05 vs the NC group, ^&^*P* < 0.05 vs the Hypoxia group, and ^#^*P* < 0.05 vs the Hyp + rosiglitazone group, one-way ANOVA.

To explore the role of Nrf2 in regulating ferroptosis by PPARγ, rosiglitazone and si-Nrf2 intervention were performed. Nrf2 was lowly expressed in the Hypoxia at the gene and protein levels, compared with the NC groups. Nrf2 increased after rosiglitazone intervention. Compared with the Hyp + rosiglitazone group, Nrf2 decreased in the Hyp + rosiglitazone + si-Nrf2 group (Fig. 5a). Levels of MDA, 8-OHdG, Fe(II), and lipid ROS were higher in the Hyp + rosiglitazone + si-Nrf2 group than in the Hyp + rosiglitazone. GSH and GPx4 were reversed (Figs. 5b, c, e). The level of SREBP1 was not statistically different between the Hyp + rosiglitazone + si-Nrf2 and the Hyp + rosiglitazone groups (Fig. 5d). The intervention of Nrf2 did not affect the protein levels of SREBP1. Silencing of SREBP1 had no significant effect on GPx4 expression (Fig. S4c). The proliferation activity was further tested. The si-Nrf2 reduced the promoting effect of rosiglitazone on cell proliferation. The protein levels of Cyclin D1, Bcl-2, c-Myc, and pHH3 were lower in the Hyp + rosiglitazone + si-Nrf2 group than in the Hyp + rosiglitazone group (Fig. 5f). The migratory and invasive abilities were lower in the Hyp + rosiglitazone + si-Nrf2 group than in the Hyp + rosiglitazone group (Fig. 5g). MMP-2 and MMP-9 decreased in the Hyp + rosiglitazone + si-Nrf2 compared to the Hyp + rosiglitazone group. The expression of TIMP-1 and TIMP-2 was the opposite. Thus, inhibition of Nrf2 reversed hypoxia-induced ferroptosis by rosiglitazone.

### Overexpression of GPx4 inhibits ferroptosis and promotes proliferation, migration and invasion activity

We verified the effect of silencing SREBP1 on lipid synthesis induced by PPAPγ activation. The results showed that rosiglitazone combined with SREBP1 silencing treatment further reduced the expression of FASN, ACC1, and SCD1 compared with rosiglitazone intervention alone (Fig. S4d).

**Fig. 6:**
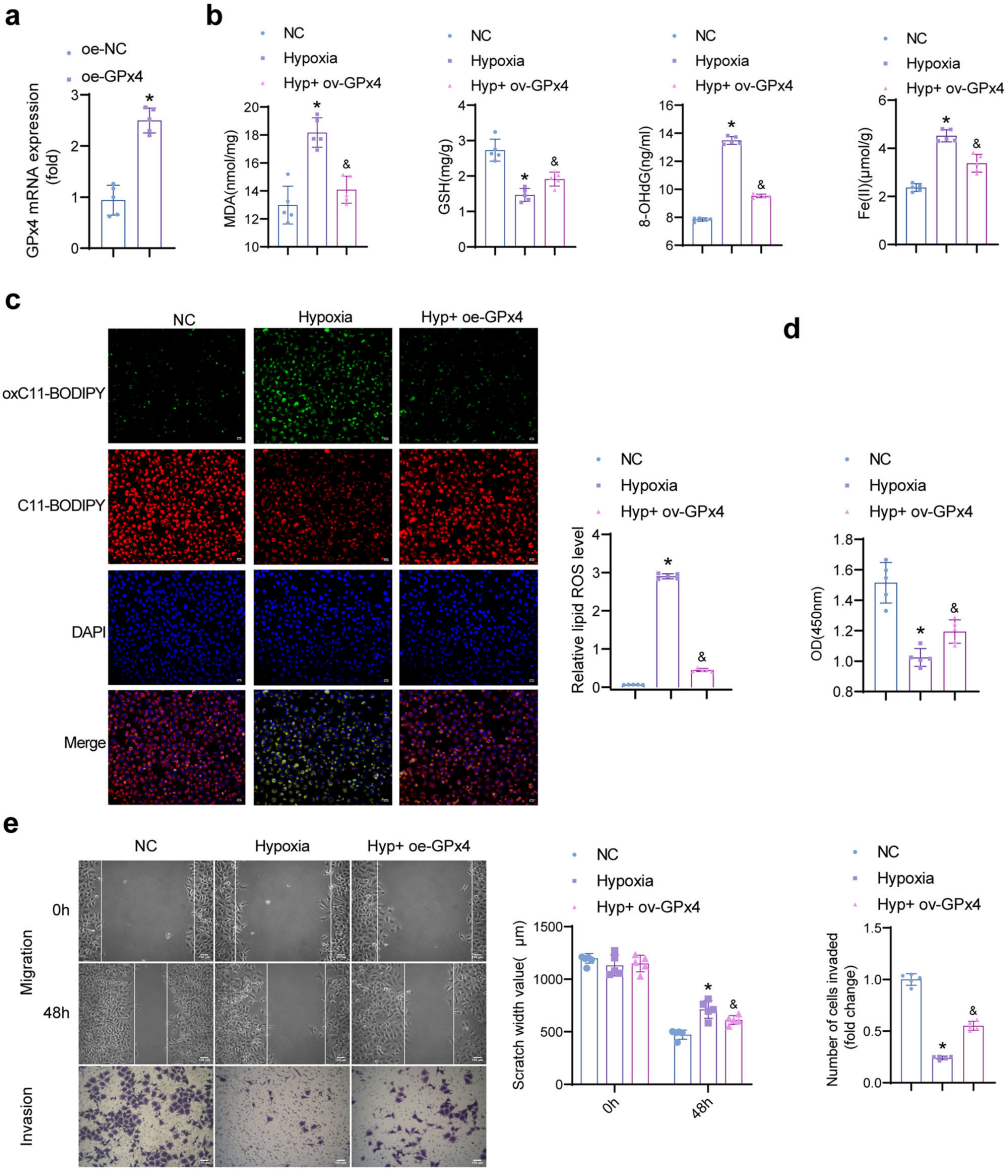
Regulating GPx4 inhibits ferroptosis and promotes proliferation and migration activity. **a** Expression of GPx4 mRNA. Data represent the mean ± SEM. *P < 0.05 vs the oe-NC group, unpaired *t*-test. **b** Levels of MDA, GSH, 8-OHdG, Fe(II). (*n* = 5). **c** Levels of lipid ROS. (200×, *n* = 5). **d** Proliferation activity. (*n* = 5). **e** Wound healing and Transwell assay for cell migration and invasion activity. (100×, *n* = 5). Data represent the mean ± SEM. *P < 0.05 vs the NC group, and ^&^*P* < 0.05 vs the Hypoxia group, one-way ANOVA.

Next, The regulation of ferroptosis by GPx4 was further characterized in HTR-8/Svneo cells. After the intervention of oe-GPx4, the expression of GPx4 mRNA was verified by qRT-PCR. GPx4 was highly expressed in oe-GPx4, compared to the oe-NC group (Fig. 6a). MDA, 8-OHdG, and Fe(II) levels were lower in the Hyp + oe-GPx4 group than in the Hypoxia (Fig. 6b). The level of GSH was the opposite. After overexpression of GPx4, lipid ROS levels decreased (Fig. 6c). Cell proliferation, migration, and invasion activities were higher in the Hyp + oe-GPx4 than in the Hypoxia groups (Fig. 6d, e). It was shown that overexpression of GPx4 could inhibit ferroptosis and promote cell development.

### PPARγ agonist rosiglitazone improves disease progression in PE rats

**Fig. 7:**
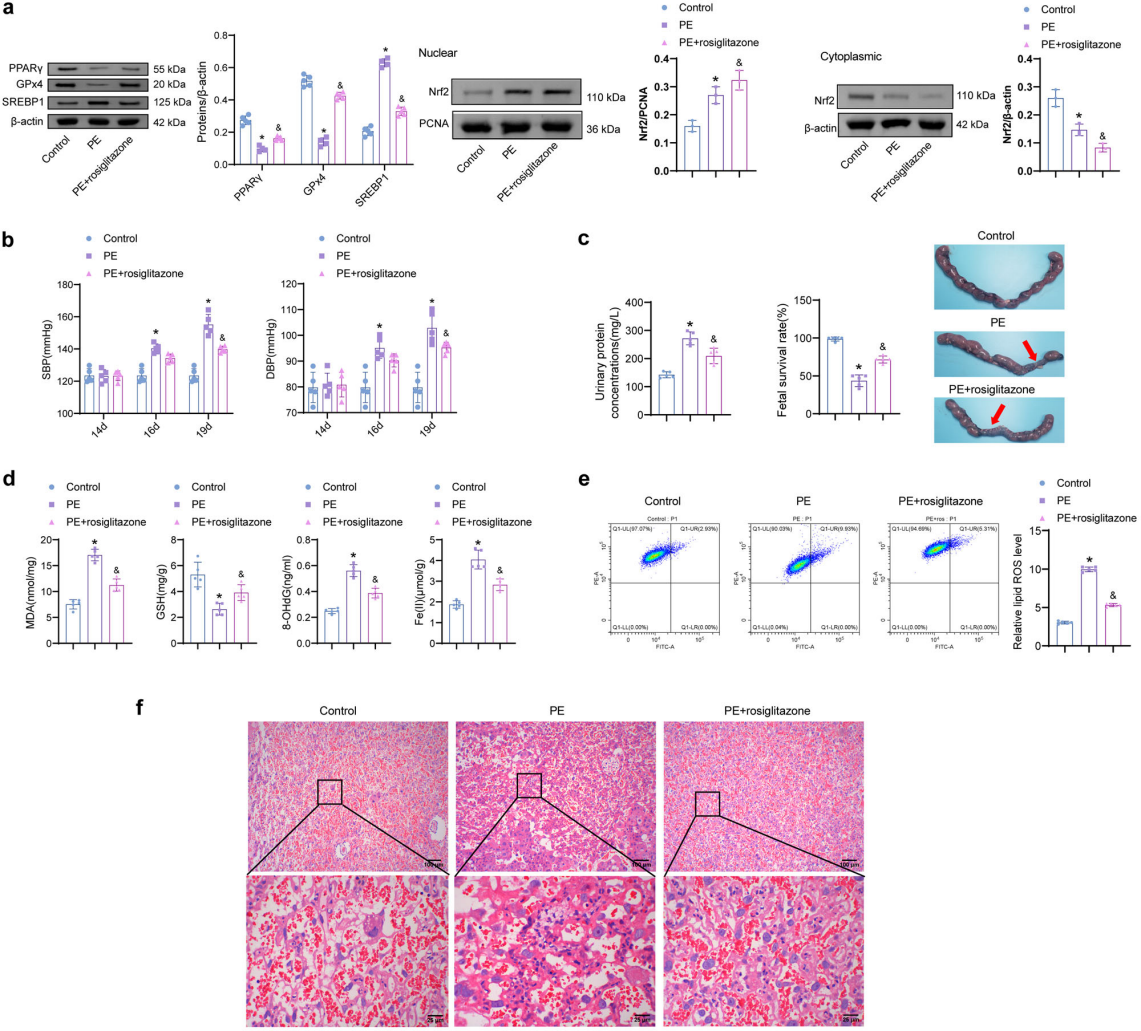
Rosiglitazone eases the development of PE. **a** PPARγ, Nrf2 (nuclear and cytoplasmic), SREBP1, and GPX4 protein expression. (*n* = 3). **b** The SBP and DBP levels on days 14, 16, and 19 of gestation. **c** The rat urine protein concentration and fetal survival at day 19. **d**, **e** Levels of MDA, GSH, 8-OHdG, Fe(II), and lipid ROS. (*n* = 5). Data represent the mean ± SEM. *P < 0.05 vs the Control group, ^&^*P* < 0.05 vs the PE group, one-way ANOVA and two-way ANOVA. **f** H&E staining. (100× and 400×).

Furthermore, rosiglitazone was used to intervene in SD pregnant rats to verify the effect of PPARγ agonist on PE. The rat placenta was collected for Western blot analysis. The PPARγ, Nrf2 (nuclear), SREBP1, and GPX4 protein levels were dysregulated in PE rats. Compared with the PE group, PPARγ, Nrf2 (nuclear), and GPX4 were increased in the PE + rosiglitazone group, and SREBP1 decreased (Fig. 7a). On days 14, 16, and 19 of gestation, the SBP and DBP levels were identified (Fig. 7b). On days 16 and 19, the SBP and DBP of the rats in the PE group were significantly higher than those in the Control group. On day 19, the SBP and DBP in the PE + rosiglitazone group were significantly lower than those in the PE. The urine protein concentration and fetal survival rate of rats were further detected. The urinary protein in the PE group was significantly increased, and the fetal survival rate decreased (Fig. 7c). The urinary protein concentration of rats in the PE + rosiglitazone group was lower than that in the PE group, and the fetal survival rate was just the opposite. MDA, 8-OHdG, and Fe(II) levels were reduced in the PE + rosiglitazone group compared with the PE group (Fig. 7d). GSH was higher in the PE + rosiglitazone than in the PE groups, and levels of lipid ROS were opposite (Fig. 7e). The placental changes were observed by H&E staining. There was obvious inflammatory infiltration in the placenta of the PE group, which was alleviated by rosiglitazone intervention (Fig. 7f).

## Discussion

The main function of the placenta is to exchange nutrients and oxygen between the mother and the fetus. Impaired neovascularization and increased placental vascular resistance in PE patients lead to local hypoxia or ischemia of the placenta, resulting in excessive ROS production^30^. Ferroptosis is a novel form of programmed cell death caused by highly iron-dependent lipid peroxidative damage^31^. In this study, lipid peroxidation-related MDA, 8-OHdG, and ROS expression increased with PE severity while GSH decreased. These suggested that lipid peroxidation occurs in the placental tissue of PE patients. The levels of ferroptosis-related indicators (GPx4 and Fe(II)) were further identified, and GPx4 was significantly decreased in severe PE, while Fe(II) content increased with the deepening of PE. It was consistent with previous findings that ferroptosis occurred in the placental tissue of PE^25^.

Previous studies have reported that PPARγ and NRF2 play a key role in reducing lipid peroxidation and ferroptosis in different diseases, including cancer, intracerebral hemorrhage, and diabetic cardiomyopathy^32–34^. Rosiglitazone, an agonist of PPARγ, could concentration-dependently upregulate the expression of Nrf2 and GPx4 and downregulate SREBP1, reversing the hypoxia-induced dysregulation. Furthermore, we found a protein interaction between PPARγ/Nrf2 and PPARγ/SREBP1 by Co-IP assay. After regulating PPARγ and RXRα, the expression of Nrf2 and SREBP1 changed. Nrf2 is a ubiquitous pleiotropic transcription factor and a major homeostatic regulator of intracellular stress^35–37^. GPx4, which initiates ferroptosis, belongs to the downstream target genes of Nrf2^38^. Furthermore, Nrf2 is involved in the downstream regulation of PPARγ, and PPARγ agonists enhance PPARγ expression in an Nrf2-dependent manner^28^. After regulating the expression of PPARγ and Nrf2, we found that the levels of ferroptosis-related indicators changed. Silencing Nrf2 reversed the inhibitory effect of rosiglitazone on ferroptosis. Meanwhile, SREBP1 associated with lipogenesis was not regulated by si-Nrf2. Activation of the transcription factor SREBP1 promotes the expression of adipogenic genes^39,40^. SREBP1 silencing had no significant effect on GPx4. It was confirmed that in hypoxia-induced HTR-8/SVneo cells, PPARγ/Nrf2 signaling affects ferroptosis by regulating lipid peroxidation, but not by suppressing lipid synthesis via SREBP1^41^. These were consistent with previous studies that Nrf2 signaling regulated ferroptosis by mediating lipid peroxidation^38^. Thus, we reasoned that PPARγ may regulate ferroptosis through Nrf2 in PE.

Ferroptosis is associated with proliferation, migration and invasion^42,43^. GPx4 is a key ferroptosis regulator, converting toxic lipid peroxides into nontoxic lipid alcohols^44^. Inactivation of GPx4 could promote the accumulation of lipid peroxidation, leading to ferroptosis^45^. Our results suggested that silencing Nrf2 could inhibit the expression of GPx4. Previous studies have shown that GPx4 is a transcriptional target of Nrf2^46,47^. Under exogenous stimulation, the residues of Keap1 are changed, leading to the nuclear translocation of Nrf2, which binds to the antioxidant-responsive element (ARE) and promotes the expression of GPx4^48–50^. Furthermore, GPx4 can regulate proliferation and migration in addition to its antioxidant capacity^51^. Similar to our results, overexpression of GPx4 promoted cell proliferation, migration, and invasive activity. Thus, the balance between ferroptosis and proliferation, migration and invasion may be regulated by the expression of ferroptosis-related genes.

However, the mechanism of targeting between PPAR and Nrf2 in this study is an open question, and combined protein site-directed mutagenesis and Co-IP experiments are needed to further analyze their possible binding sites. The low level of HDL in PE is an observed marker. Previous results also found that HDL levels were reduced in patients with PE^52,53^. Compared with healthy pregnant women, PE patients have abnormal lipid metabolism. We hypothesized that decreased HDL levels may be associated with the pathogenesis of PE. However, the mechanism remains to be further studied. Whether Nrf2 mediates other processes of lipid metabolism, such as lipid synthesis, breakdown, and transport, is limited. In addition, the question that Nrf2 expression can be regulated by other proteins could not be perfectly addressed in this study. Nrf2 expression is regulated by a variety of proteins^54^. The upstream and downstream regulation mechanism is a complex and huge mechanism network, which needs to be further explored in combination with multi-omics.

The study confirmed the occurrence of ferroptosis in the PE placenta. The PPARγ agonist rosiglitazone could reverse hypoxia-induced ferroptosis. PPARγ affected ferroptosis by regulating lipid peroxidation via Nrf2 signaling but not by inhibiting SREBP1 to suppress lipid synthesis. In conclusion, we found that activation of PPARγ may suppress hypoxia-induced ferroptosis through Nrf2 signaling and improve PE progression.

### Supplementary information

Supplementary Information

Description of Additional Supplementary Materials

Supplementary Data 1

Reporting Summary

## Data Availability

All data found and analyzed during this study are included in this paper and its supplementary files. The source data can be found in Supplementary Data 1.
