# Supplementary material for: PPARγ alleviates preeclampsia development by regulating lipid metabolism and ferroptosis
Source: Commun Biol. 2024 Apr 9;7:429. doi: 10.1038/s42003-024-06063-2 (PMC11004023; doi:10.1038/s42003-024-06063-2)
Supplement: Supplementary file 1 — Supplementary Information [file 42003_2024_6063_MOESM1_ESM.docx]

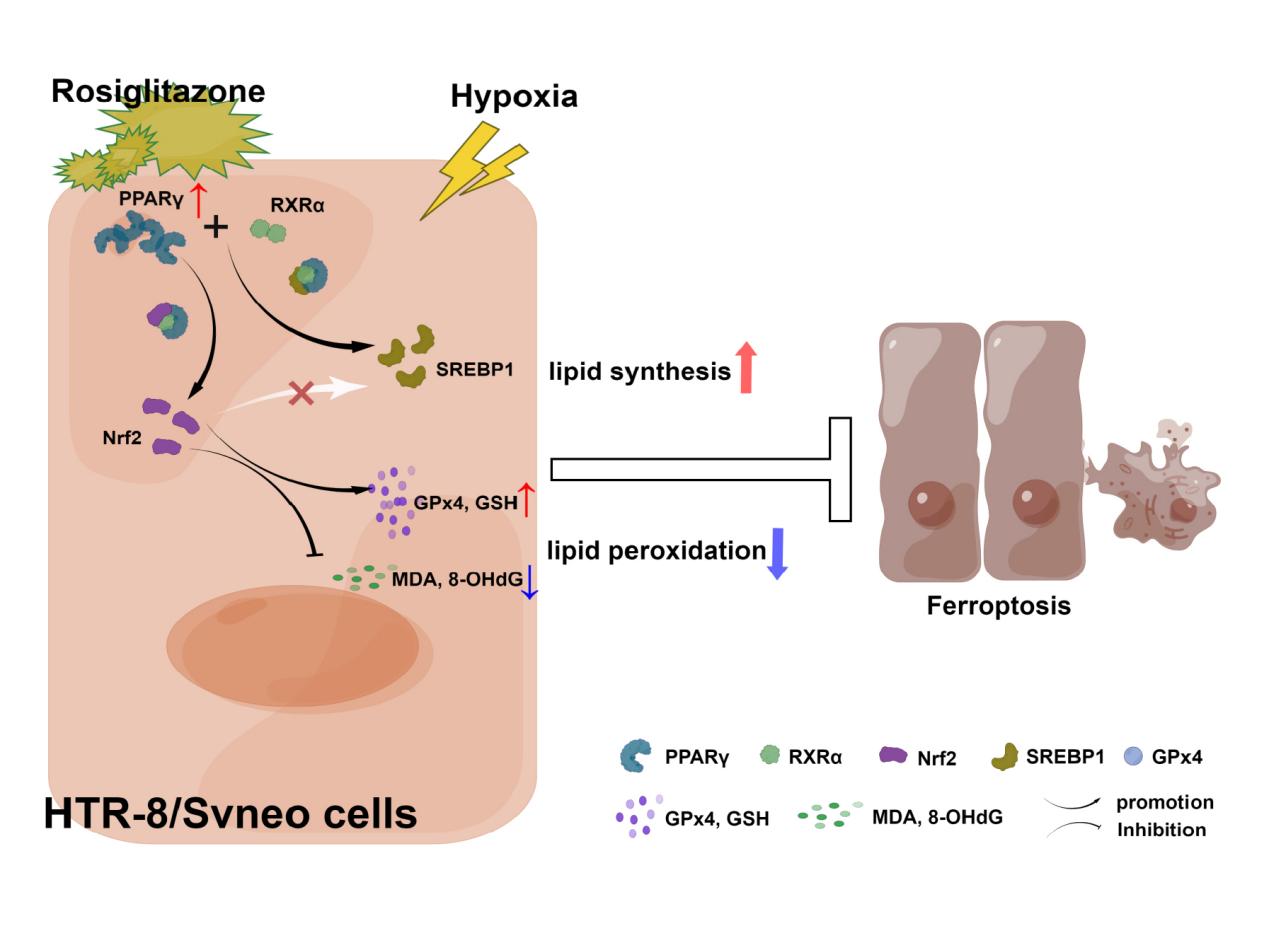


**Figure S1. The mechanical diagram of the study.** A mechanistic diagram of PPARγ signaling regulating hypoxia-induced ferroptosis in HTR-8/Svneo cells with/without rosiglitazone intervention was built by Figdraw software.


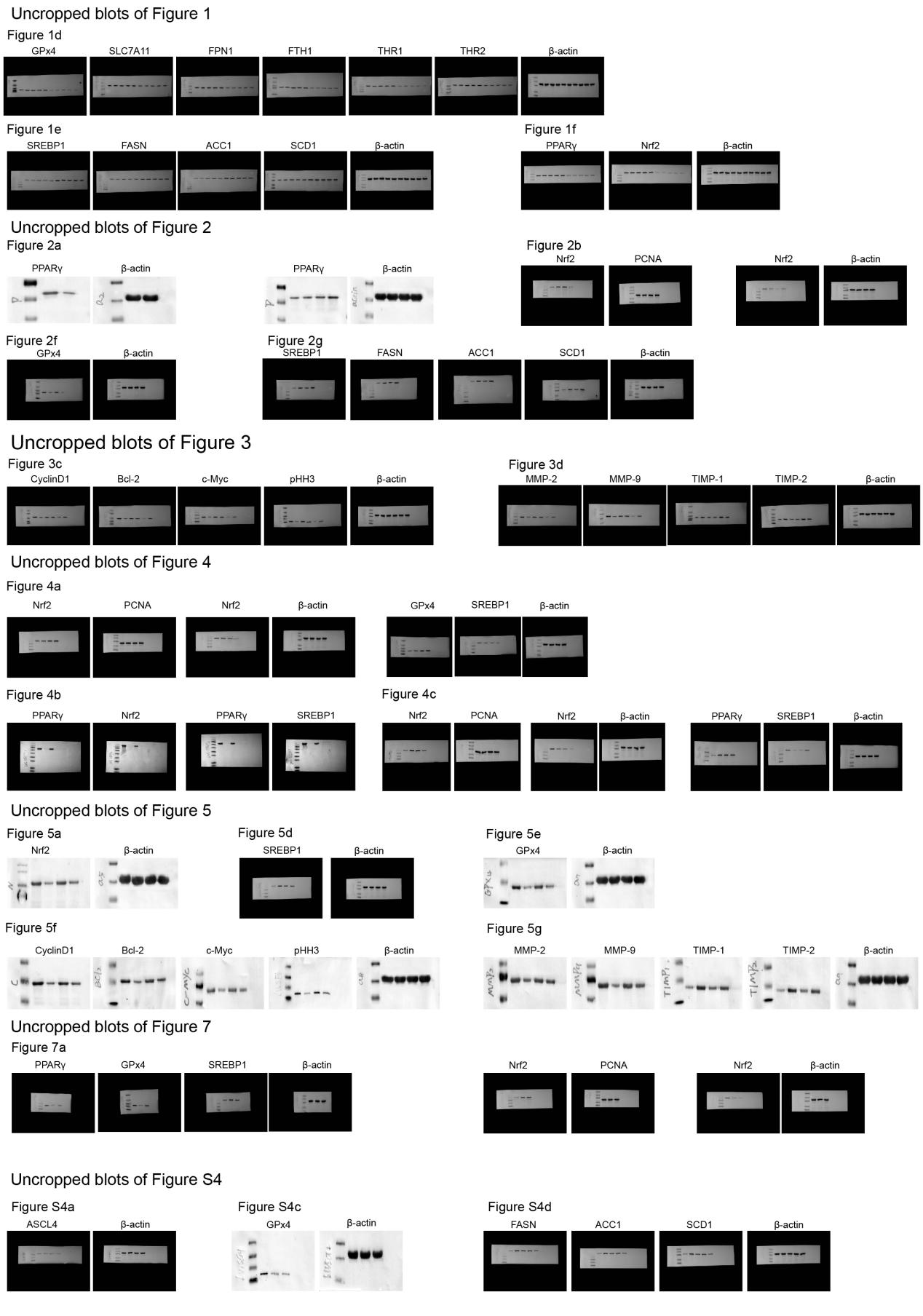


**Figure S2. Uncropped blots.**


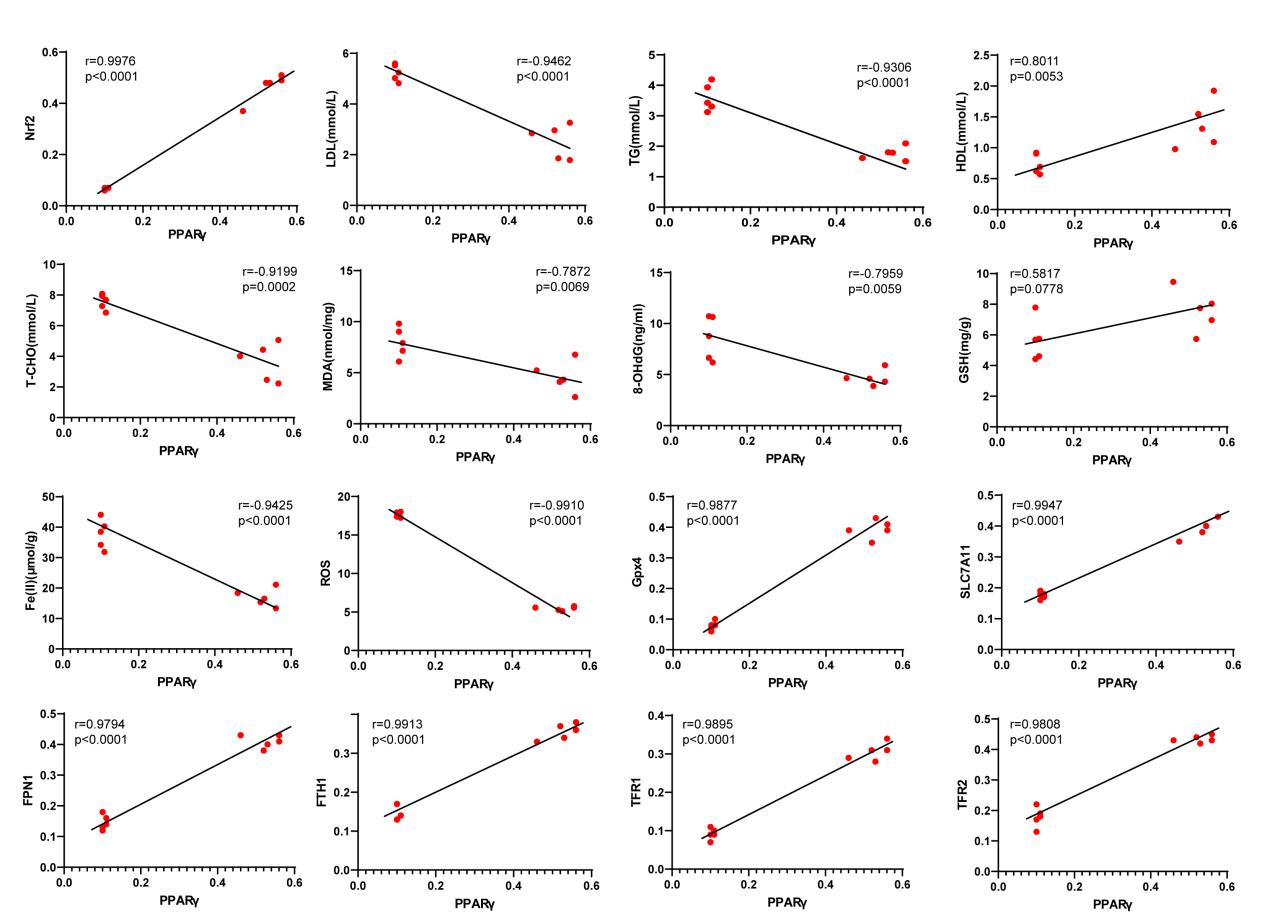


**Figure S3. Pearson correlation analysis.Correlation analysis of PPARγ with Nrf2 levels and ferroptosis-related indicators in PE was performed.**


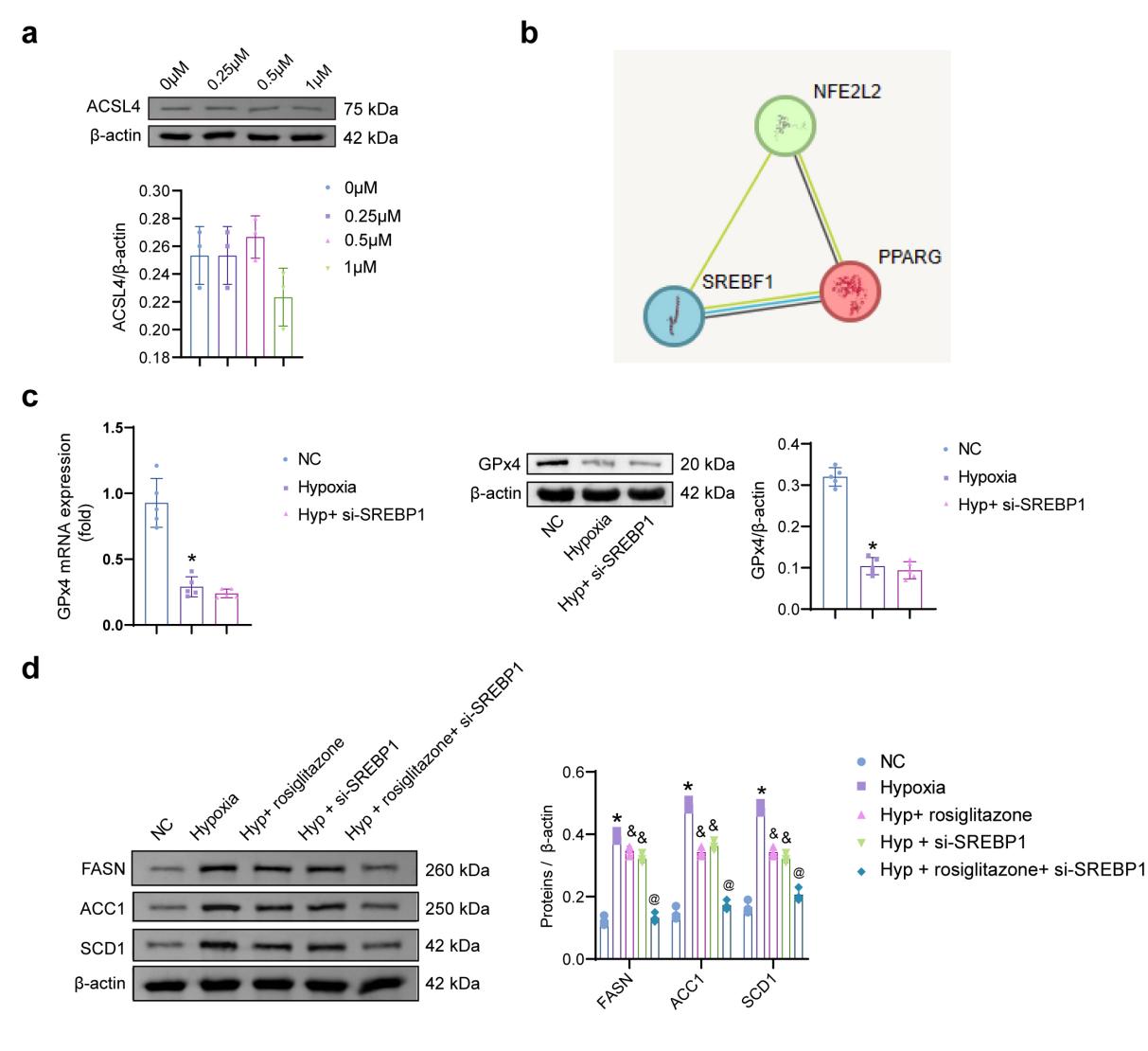


**Figure S4. Expression verification of GPx4, ACSL4, FASN, ACC1, and SCD1.** (a) GPx4 mRNA and protein levels. *P<0.05 vs the NC group, one-way ANOVA. (b) PPARγ binding to SREBP1 and Nrf2 was predicted using the online software STRING. (c) ACSL4 protein levels. (d) FASN, ACC1, and SCD1 levels. (n=3). *P<0.05 vs the NC group, &P<0.05 vs the Hypoxia group, and @P<0.05 vs the Hyp + si-SREBP1 group, two-way ANOVA.
