# Supplementary material for: PPARγ alleviates preeclampsia development by regulating lipid metabolism and ferroptosis
Source: Commun Biol. 2024 Apr 9;7:429. doi: 10.1038/s42003-024-06063-2 (PMC11004023; doi:10.1038/s42003-024-06063-2)
Supplement: Supplementary file 4 — Reporting Summary [file 42003_2024_6063_MOESM4_ESM.pdf]

nature portfolio

Corresponding author(s): YD
Manuscript number: COMMSBIO-23-2815

Last updated by author(s): Mar 12, 2024

Reporting Summary

Nature Portfolio wishes to improve the reproducibility of the work that we publish. This form provides structure for consistency and transparency in reporting. For further information on Nature Portfolio policies, see our Editorial Policies and the Editorial Policy Checklist.

Statistics

For all statistical analyses, confirm that the following items are present in the figure legend, table legend, main text, or Methods section.

n/a	Confirmed	
☒	☐	The exact sample size (n) for each experimental group/condition, given as a discrete number and unit of measurement
☒	☐	A statement on whether measurements were taken from distinct samples or whether the same sample was measured repeatedly
☐	☒	The statistical test(s) used AND whether they are one- or two-sided<br>*Only common tests should be described solely by name; describe more complex techniques in the Methods section.*
☐	☒	A description of all covariates tested
☐	☒	A description of any assumptions or corrections, such as tests of normality and adjustment for multiple comparisons
☐	☒	A full description of the statistical parameters including central tendency (e.g. means) or other basic estimates (e.g. regression coefficient) AND variation (e.g. standard deviation) or associated estimates of uncertainty (e.g. confidence intervals)
☐	☒	For null hypothesis testing, the test statistic (e.g. F, t, r) with confidence intervals, effect sizes, degrees of freedom and P value noted<br>*Give P values as exact values whenever suitable.*
☒	☐	For Bayesian analysis, information on the choice of priors and Markov chain Monte Carlo settings
☒	☐	For hierarchical and complex designs, identification of the appropriate level for tests and full reporting of outcomes
☒	☐	Estimates of effect sizes (e.g. Cohen's d, Pearson's r), indicating how they were calculated

Our web collection on statistics for biologists contains articles on many of the points above.

Software and code

Policy information about availability of computer code

Data collection	N/A
Data analysis	N/A

For manuscripts utilizing custom algorithms or software that are central to the research but not yet described in published literature, software must be made available to editors and reviewers. We strongly encourage code deposition in a community repository (e.g. GitHub). See the Nature Portfolio guidelines for submitting code & software for further information.

Data

Policy information about availability of data

All manuscripts must include a data availability statement. This statement should provide the following information, where applicable:

- Accession codes, unique identifiers, or web links for publicly available datasets
- A description of any restrictions on data availability
- For clinical datasets or third party data, please ensure that the statement adheres to our policy

All data found and analyzed during this study are included in this paper and its supplementary files.

Research involving human participants, their data, or biological material

Policy information about studies with human participants or human data. See also policy information about sex, gender (identity/presentation), and sexual orientation and race, ethnicity and racism.

Reporting on sex and gender	The subjects' clinical baseline information is shown in Table 1.
Reporting on race, ethnicity, or other socially relevant groupings	The subjects' clinical baseline information is shown in Table 1.
Population characteristics	The subjects' clinical baseline information is shown in Table 1.
Recruitment	N/A
Ethics oversight	The subjects' clinical baseline information is shown in Table 1.

Note that full information on the approval of the study protocol must also be provided in the manuscript.

Field-specific reporting

Please select the one below that is the best fit for your research. If you are not sure, read the appropriate sections before making your selection.

☒ Life sciences ☐ Behavioural & social sciences ☐ Ecological, evolutionary & environmental sciences

For a reference copy of the document with all sections, see nature.com/documents/nr-reporting-summary-flat.pdf

Life sciences study design

All studies must disclose on these points even when the disclosure is negative.

Sample size	Clinical sample: This was the largest sample size the authors could collect to meet the criteria.<br>Animall sample: The sample size of each group was set according to the reference (PMID: 33835684).
Data exclusions	Clinical sample exclusion criteria were patients with cardiovascular disease, diabetes, metabolic syndrome, infection, congenital malformations, and chromosomal abnormalities (number and/or structure).
Replication	At least three biological replicates were performed for each experiment.
Randomization	Clinical sample: The inclusion criteria for the Normal were normotensive during pregnancy, term pregnancy, no history of chronic metabolic disease or any pathology that may involve disturbances in lipid or carbohydrate metabolism, and no complications during pregnancy. PE was diagnosed and classified according to the criteria provided by the ISSHP.
Blinding	Investigators were blinded to group assignments during data collection and analysis.

Reporting for specific materials, systems and methods

We require information from authors about some types of materials, experimental systems and methods used in many studies. Here, indicate whether each material, system or method listed is relevant to your study. If you are not sure if a list item applies to your research, read the appropriate section before selecting a response.

Materials & experimental systems

n/a	Involved in the study	
☐	☒	Antibodies
☐	☒	Eukaryotic cell lines
☒	☐	Palaeontology and archaeology
☐	☒	Animals and other organisms
☐	☒	Clinical data
☒	☐	Dual use research of concern
☒	☐	Plants

Methods

n/a	Involved in the study	
☒	☐	ChIP-seq
☐	☒	Flow cytometry
☒	☐	MRI-based neuroimaging

Antibodies

Antibodies used	PPARγ (16643-1-AP,Polyclonal, proteintech);

Antibodies used	Nrf2 (16396-1-AP, Polyclonal, proteintech);<br>SREBP1 (14088-1-AP, Polyclonal, proteintech);<br>FASN (10624-2-AP, Polyclonal, proteintech);<br>ACC1 (21923-1-AP, Polyclonal, proteintech);<br>GPx4 (67763-1-Ig, Monoclonal, proteintech);<br>SLC7A11 (26864-1-AP, Polyclonal, proteintech);<br>FPN1 (26601-1-AP, Polyclonal, proteintech);<br>FTH1 (10727-1-AP, Polyclonal, proteintech);<br>TFR1 (66180-1-Ig, Monoclonal, proteintech);<br>CyclinD1 (60186-1-Ig , Monoclonal, proteintech);<br>BCL2 (12789-1-AP, Polyclonal, proteintech);<br>C-Myc (10828-1-AP, Polyclonal, proteintech);<br>pHH3 (ab5176, Polyclonal, abcam);<br>MMP2 (10373-2-AP, Polyclonal, proteintech);<br>MMP9 (10375-2-AP, Polyclonal, proteintech);<br>TIMP-1 (16644-1-AP, Polyclonal, proteintech);<br>TIMP-2 (17353-1-AP, Polyclonal, proteintech);<br>β-actin (66009-1-Ig, Monoclonal, proteintech);<br>SCD1 (ab19862, monoclonal, abcam);<br>TFR2 (ab80194, polyclonal, abcam).
Validation	PPARγ Photoacoustic molecular imaging-escorted adipose photodynamic-browning synergy for fighting obesity with virus-like complexes. https://www.ptgcn.com/products/PPARG-Antibody-16643-1-AP.htm<br>Nrf2 Nucleolar HEATR1 upregulated by mTORC1 signaling promotes hepatocellular carcinoma growth by dominating ribosome biogenesis and proteome homeostasis https://www.ptgcn.com/products/NFE2L2,NRF2-Antibody-16396-1-AP.htm#publications<br>SREBP1 CircACC1 Regulates Assembly and Activation of AMPK Complex under Metabolic Stress. https://www.ptgcn.com/products/SREBF1-Antibody-14088-1-AP.htm#publications<br>FASN Elevation of JAML Promotes Diabetic Kidney Disease by Modulating Podocyte Lipid Metabolism. https://www.ptgcn.com/products/FASN-Antibody-10624-2-AP.htm#publications<br>ACC1 Mannose antagonizes GSDME-mediated pyroptosis through AMPK activated by metabolite GlcNAc-6P https://www.ptgcn.com/products/ACACA-Antibody-21923-1-AP.htm#publications<br>GPx4 Liberation of daidzein by gut microbial β-galactosidase suppresses acetaminophen-induced hepatotoxicity in mice https://www.ptgcn.com/products/GPX4-Antibody-67763-1-Ig.htm#publications<br>SLC7A11 Mitochondria-localized cGAS suppresses ferroptosis to promote cancer progression https://www.ptgcn.com/products/xCT-Antibody-26864-1-AP.htm#publications<br>FPN1 NCOA4-mediated ferritinophagy is involved in ionizing radiation-induced ferroptosis of intestinal epithelial cells. https://www.ptgcn.com/products/SLC40A1-Antibody-26601-1-AP.htm#publications<br>FTH1 Lactate modulates iron metabolism by binding soluble adenylyl cyclase https://www.ptgcn.com/products/FTL-Antibody-10727-1-AP.htm#publications<br>TFR1 Super-enhancer-driven MLX mediates redox balance maintenance via SLC7A11 in osteosarcoma https://www.ptgcn.com/products/CD71-Antibody-66180-1-Ig.htm#publications<br>CyclinD1 TRIM15 and CYLD regulate ERK activation via lysine-63-linked polyubiquitination. https://www.ptgcn.com/products/CCND1-Antibody-60186-1-Ig.htm#publications<br>BCL2 Targeting CRL4 suppresses chemoresistant ovarian cancer growth by inducing mitophagy https://www.ptgcn.com/products/BCL2-Antibody-12789-1-AP.htm#publications<br>C-Myc Overexpression of CIP2A is associated with poor prognosis in multiple myeloma. https://www.ptgcn.com/products/MYC-Antibody-10828-1-AP.htm#publications<br>pHH3 MTH1 as a target to alleviate T cell driven diseases by selective suppression of activated T cells https://www.abcam.com/products/primary-antibodies/histone-h3-phospho-s10-antibody-ab5176.html<br>MMP2 Unspliced XBP1 Confers VSMC Homeostasis and Prevents Aortic Aneurysm Formation via FoxO4 Interaction. https://www.ptgcn.com/products/MMP2-Antibody-10373-2-AP.htm#publications<br>MMP9 Regulator of Calcineurin 1 Gene Isoform 4, Downregulated in Hepatocellular Carcinoma, Prevents Proliferation, Migration, and Invasive Activity of Cancer Cells and Growth of Orthotopic Tumors by Inhibiting Nuclear Translocation of NFAT1. https://www.ptgcn.com/products/MMP9-Antibody-10375-2-AP.htm<br>TIMP-1 Addiction to Golgi-resident PI4P synthesis in chromosome 1q21.3-amplified lung adenocarcinoma cells. https://www.ptgcn.com/products/TIMP1-Antibody-16644-1-AP.htm#publications<br>TIMP-2 RAB37 Hypermethylation Regulates Metastasis and Resistance to Docetaxel-Based Induction Chemotherapy in Nasopharyngeal Carcinoma. https://www.ptgcn.com/products/TIMP2-Antibody-17353-1-AP.htm#publications<br>β-actin Structure of Semliki Forest virus in complex with its receptor VLDLR https://www.ptgcn.com/products/Pan-Actin-Antibody-66009-1-Ig.htm#publications<br>SCD1 Targeting of the Lipid Metabolism Impairs Resistance to BRAF Kinase Inhibitor in Melanoma https://www.abcam.com/products/primary-antibodies/scd1-antibody-cde10-ab19862.html<br>TFR2 SEC23B Loss-of-Function Suppresses Hepcidin Expression by Impairing Glycosylation Pathway in Human Hepatic Cells https://www.abcam.com/products/primary-antibodies/transferrin-receptor-2tfr2-antibody-ab80194.html

Eukaryotic cell lines

Policy information about cell lines and Sex and Gender in Research

Cell line source(s)	HTR-8/SVneo cells were obtained from Shanghai Zhong Qiao Xin Zhou Biotechnology Co., Ltd. Cells were cultured in RPMI-1640 medium containing 10% fetal bovine serum (FBS) and 1% penicillin/streptomycin. Cells were grown at 37°C, 5% CO2.
Authentication	HTR-8/SVneo cells were obtained from Shanghai Zhong Qiao Xin Zhou Biotechnology Co., Ltd. Cells were cultured in

Authentication	RPMI-1640 medium containing 10% fetal bovine serum (FBS) and 1% penicillin/streptomycin. Cells were grown at 37°C, 5% CO_2.
Mycoplasma contamination	NO
Commonly misidentified lines (See ICLAC register)	HTR-8

Animals and other research organisms

Policy information about studies involving animals; ARRIVE guidelines recommended for reporting animal research, and Sex and Gender in Research

Laboratory animals	Rats
Wild animals	No wild animals were involved in this study.
Reporting on sex	Female rats: Pregnant rats were subjected to PE induction and analysis.
Field-collected samples	N/A
Ethics oversight	The Animal Ethical and Welfare Committee, the Second Xiangya Hospital, Central South University, China (2021806).

Note that full information on the approval of the study protocol must also be provided in the manuscript.

Clinical data

Policy information about clinical studies

All manuscripts should comply with the ICMJE guidelines for publication of clinical research and a completed CONSORT checklist must be included with all submissions.

Clinical trial registration	The Second Xiangya Hospitol of Central South University Ethics Committee (2020-584).
Study protocol	the inclusion criteria for the Normal were normotensive during pregnancy, term pregnancy, no history of chronic metabolic disease or any pathology that may involve disturbances in lipid or carbohydrate metabolism, and no complications during pregnancy. Exclusion criteria for PE were patients with cardiovascular disease, diabetes, metabolic syndrome, infection, congenital malformations, and chromosomal abnormalities (number or structure). The subjects' clinical baseline information is shown in Table 1.
Data collection	No clinical data were collected. Clinical samples were collected in the Second Xiangya Hospital of Central South University from September 2021 to November 2021.
Outcomes	N/A

Plants

Seed stocks	N/A
Novel plant genotypes	N/A
Authentication	N/A

Flow Cytometry

Plots

Confirm that:

☒ The axis labels state the marker and fluorochrome used (e.g. CD4-FITC).

☒ The axis scales are clearly visible. Include numbers along axes only for bottom left plot of group (a 'group' is an analysis of identical markers).

☒ All plots are contour plots with outliers or pseudocolor plots.

☒ A numerical value for number of cells or percentage (with statistics) is provided.

Methodology

Sample preparation	After fresh tissue was washed 3 times with 5 ml PBS, the tissue was minced to a paste with ophthalmic scissors. 5 ml of collagenase Type I was added to digest for 40 min at 37°C on a shaker. 5ml complete Dulbecco's modified eagle medium (DMEM) was added to stop digestion. The cell suspension was collected and centrifuged at 1500 rpm for 5 min to obtain cell pellets. After the cells were resuspended in 5 ml of erythrocyte lysate, the samples were allowed to stand at room temperature for 5 min, and then centrifuged at 1500 rpm for 5 min to obtain cell pellets. After resuspending and washing with 5 ml PBS, 2 ml of DMEM medium was added.
Instrument	Ultra-clean workbench Beijing YATELON YT-CJ-2NBDirectly heated carbon dioxide incubator Shanghai Sunten Instrument DH-160IInverted Biological Microscope DSZ2000XLow-speed centrifuge SL02Flow meter Beckman A00-1-1102
Software	Ultra-clean working table Beijing YATELON YT-CJ-2NB;Directly heated carbon dioxide incubator Shanghai Santo Instruments DH-160I;Inverted biological microscope Beijing Zhongxian Hengye Instrument DSZ2000X;Low-speed centrifuge Zhixin Instruments SL02;Flow meter Beckman A00-1-1102.
Cell population abundance	N/A
Gating strategy	N/A

☒ Tick this box to confirm that a figure exemplifying the gating strategy is provided in the Supplementary Information.
